# Acromegaly and Cardiovascular Disease: Associated Cardiovascular Risk Factors, Cardiovascular Prognosis, and Therapeutic Impact

**DOI:** 10.3390/jcm14061906

**Published:** 2025-03-12

**Authors:** Pedro Iglesias

**Affiliations:** 1 Department of Endocrinology and Nutrition, Hospital Universitario Puerta de Hierro Majadahonda, Calle Joaquín Rodrigo, 1, 28222 Majadahonda, Madrid, Spain; piglo65@gmail.com; 2 Instituto de Investigación Sanitaria Puerta de Hierro Segovia de Arana, 28222 Majadahonda, Madrid, Spain

**Keywords:** acromegaly, diabetes, insulin resistance, dyslipidemia, hypertension, endothelial dysfunction, cardiovascular disease, arrhythmia, acromegalic cardiomyopathy

## Abstract

Acromegaly is a chronic disease characterized by the excessive production of growth hormone (GH), resulting in elevated levels of insulin-like growth factor-1 (IGF-1). It is associated with a significantly increased risk of cardiovascular complications, including arrhythmias and acromegalic cardiomyopathy, which are major contributors to morbidity and mortality in patients with acromegaly. Providing a comprehensive analysis of the cardiovascular risk factors and cardiovascular diseases associated with acromegaly, as well as examining their impact on prognosis and therapeutic strategies that can improve cardiovascular health in these patients, is key to understanding the magnitude of the problem and optimizing clinical management. The presence of traditional cardiovascular risk factors such as diabetes (with a prevalence ranging from 22.3% to 76.8%), hypertension (from 18% to 77%), and dyslipidemia (up to 61%) is worsened by disease activity and duration, increasing the likelihood of adverse cardiovascular events. Early diagnosis and effective treatment are critical to alleviating these complications, as the normalization of GH and IGF-1 levels can improve cardiovascular prognosis. In addition, comprehensive management, including the control of cardiovascular risk factors and regular assessment of cardiac function, is essential. Data suggest that with appropriate treatment, the incidence of myocardial infarction and stroke can be similar to that in the general population. In conclusion, paying careful attention to cardiovascular complications in patients with acromegaly will not only enhance their quality of life, but may also increase their life expectancy through the effective management of comorbidities associated with this disease.

## 1. Introduction

Acromegaly is a chronic endocrine disease characterized by the excessive production of growth hormone (GH), which causes an increase in plasma concentrations of insulin-like growth factor-1 (IGF-1), triggering various morphological alterations and systemic complications [1,2]. The prevalence of acromegaly ranges between 40 and 104 cases per million population, and it has an annual incidence of between 3 and 8 new cases per million [3,4]. Other studies have reported an even higher prevalence, with figures ranging from 100 to 1043 cases per million population [4,5,6,7], suggesting that a significant number of patients may be undiagnosed or go undetected. Acromegaly is usually diagnosed more frequently in middle-aged adults, with an average patient age of 40 years, affecting men and women equally [8]. In most cases (>95%), acromegaly is caused by a GH-secreting pituitary adenoma and, in very rare cases, can be due to the ectopic secretion of GH-releasing hormone (GHRH), resulting in pituitary hyperplasia [1].

The pathophysiology of acromegaly extends beyond the direct effects of elevated growth hormone and IGF-1 levels; it also encompasses a range of pathological changes that increase the risk of serious health complications. The dysregulated metabolism and altered hormonal signals associated with acromegaly frequently lead to insulin resistance, dyslipidemia, and hypertension, all of which can contribute to the accelerated development of cardiovascular disease. Research indicates that patients with acromegaly have a higher incidence of metabolic syndrome—a cluster of conditions that increase the risk of heart disease, stroke, and diabetes—highlighting the urgent need for the careful monitoring and management of these conditions [6,7,9].

Acromegaly has a significant impact on the cardiovascular system, and cardiovascular complications are one of the main causes of morbidity and mortality in patients with acromegaly [9,10,11,12]. These include hypertension, acromegalic cardiomyopathy, atherosclerosis and coronary artery disease, valvular disease, arrhythmias, and sudden cardiac death. Consequently, it is critical to recognize cardiovascular complications in acromegaly early and manage them appropriately to improve the prognosis and quality of life of patients [13]. The normalization of GH and IGF-1 levels by surgical and/or medical treatment can at least partially reverse cardiovascular alterations, although in advanced cases, some complications may persist despite biochemical control of the disease.

Therefore, it is essential to control traditional cardiovascular risk factors, such as hypertension, diabetes, obesity, and dyslipidemia, in patients with acromegaly, and to conduct a comprehensive cardiovascular evaluation that includes imaging studies and the periodic monitoring of cardiac function to detect and treat associated complications promptly [13,14,15]. The present review discusses in detail the main cardiovascular risk factors and cardiovascular diseases associated with acromegaly, as well as their prognosis and the effects of treatment. In addition to discussing these factors, the objective was to update the scientific evidence to more accurately define the relationship between acromegaly and cardiovascular disease. Current challenges and future perspectives related to cardiovascular pathology in acromegaly are also addressed. By focusing on these elements, we aim to enhance awareness and inform clinical practices that could significantly mitigate the adverse cardiovascular outcomes associated with this complex endocrine disorder.

## 2. Methods

The Medical Subject Headings (MeSH) terms used for the search included “acromegaly”, “diabetes mellitus”, “insulin resistance”, “hypertension”, “dyslipidemia”, “endothelial dysfunction”, “obesity”, “cardiovascular diseases”, “arrhythmia”, “heart failure”, “cardiomyopathy”, “stroke”, and “coronary artery disease”. These terms were used to search PubMed/Medline, Cochrane Database of Systematic Reviews, and Embase. The most relevant articles in English were selected and reviewed, with the search covering articles preferably published within the last 5–10 years and up until 31 January 2025.

The inclusion criteria comprised original studies, narrative and systematic reviews, and meta-analyses that evaluated the association between acromegaly and cardiovascular disease, as well as its risk factors and potential therapeutic approaches. Studies focusing on the pathophysiological mechanisms linking acromegaly to cardiovascular alterations were also included. Case reports, conference abstracts, and non-English articles were excluded.

## 3. Cardiovascular Risk Factors Associated with Acromegaly

**Figure 1:**
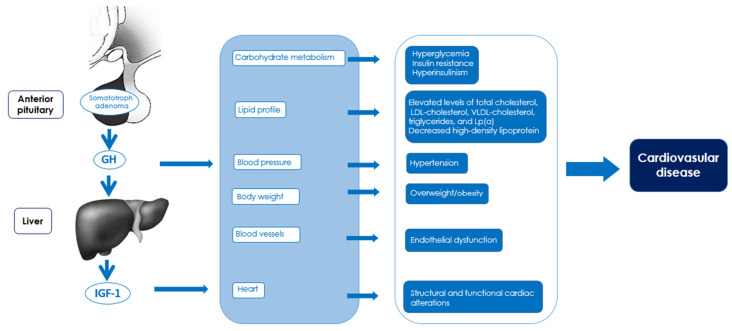
Impact of acromegaly on risk factors and pathogenic mechanisms of cardiovascular diseases.

Acromegaly negatively affects cardiovascular risk. The main effects of acromegaly on cardiovascular risk, pathogenic mechanisms, and associated clinical consequences are shown in Table 1 and Figure 1.

### 3.1. Hyperglycemia, Hyperinsulinemia, and Insulin Resistance

Excess GH in patients with acromegaly significantly affects carbohydrate metabolism, exerting a marked hyperglycemic effect. This effect is explained by several mechanisms: on the one hand, GH stimulates hepatic gluconeogenesis from non-carbohydrate sources, such as amino acids and glycerol [22]; and on the other hand, it decreases insulin sensitivity in peripheral tissues, which not only limits glucose uptake by muscle and adipose cells, but also favors the development of hyperinsulinism [23]. Beta-cell dysfunction has also been reported in acromegaly, resulting in decreased insulin secretion in response to glucose [24]. These changes make carbohydrate metabolism disorders particularly common in patients with acromegaly [25]. In this context, a prevalence of prediabetes (altered basal glycemia and glucose intolerance) and diabetes mellitus of 26–41% and 22.3–76.8%, respectively, has been reported [4,26,27,28,29]. The development of diabetes in patients with acromegaly has been associated with a family history of diabetes, obesity, advanced age, and elevated IGF-1 levels [27,28]. Patients with diabetes have been shown to be at increased risk for most comorbidities including myocardial infarction (MI) and ischemic stroke [4].

### 3.2. Dyslipidemia

Acromegaly may be associated with dyslipidemia as a result of excess GH and related insulin resistance, with the combination of both factors being responsible for the occurrence of pro-oxidant and proinflammatory atherogenic factors [30]. Dyslipidemia is highly prevalent in patients with acromegaly. A study by Romanisio et al. reported that 61.1% of these patients had dyslipidemia, regardless of GH and IGF-1 levels [31]. Compared to healthy individuals, patients with acromegaly present elevated levels of total cholesterol, low-density lipoprotein (LDL) cholesterol, very-low-density lipoprotein (VLDL) cholesterol, triglycerides, and lipoprotein(a) [Lp(a)], along with decreased levels of high-density lipoprotein (HDL) cholesterol [32]. In addition, insulin resistance in these patients is associated with elevated triglyceride levels and increased cholesteryl ester transfer protein activity. In turn, elevated GH levels independently predict increased oxidized LDL and endothelin-1 [30,33]. Similarly, excess GH has a direct effect on the distribution of LDL subfractions, favoring an increase in small, dense LDL particles, which are more atherogenic. Finally, it also reduces hepatic lipase and lipoprotein lipase activity, which contributes to the development of dyslipidemia [16,17]. All these alterations contribute directly to establishing a clinical situation of greater predisposition to the development of atherosclerotic cardiovascular disease [33]. In conclusion, patients with acromegaly have a high prevalence of dyslipidemia, driven by excess GH, insulin resistance, and alterations in lipid metabolism, which collectively increase the risk of atherosclerotic cardiovascular disease.

### 3.3. Hypertension

Patients with acromegaly have an increased risk of developing arterial hypertension, with a prevalence ranging from 18% to 77% according to different reported series [4,14,18,34]. Among the mechanisms involved are excess GH and IGF-1, which generate an expansion of extracellular fluid volume, promote sodium and water retention at the renal level, increase vascular resistance, and are associated with the possible presence of sleep apnea. Similarly, metabolic alterations, such as insulin resistance and dyslipidemia, act as relevant risk factors for hypertension. On the other hand, cardiovascular changes, such as left ventricular hypertrophy and diastolic dysfunction, directly favor its development [35]. Finally, a proinflammatory state and oxidative stress, frequent in this condition, affect endothelial function, contributing significantly to the development of hypertension [19]. In summary, patients with acromegaly have a high prevalence of arterial hypertension, driven by hormonal excess, metabolic alterations, cardiovascular changes, and endothelial dysfunction.

### 3.4. Overweight and Obesity

The prevalence of overweight and obesity is high in acromegaly and is related to disease activity. Most patients (77.51%) have a body mass index (BMI) between 25 and 35 kg/m^2^. In addition, more than 40% of patients with acromegaly have a BMI greater than 30 kg/m^2^, indicating a considerable prevalence of overweight and obesity in this group of patients [36]. In contrast to what occurs in the population with obesity, where IGF-1 levels tend to be low, a positive correlation between body mass index (BMI) and IGF-1 levels has been reported in patients with acromegaly, indicating that the higher the disease activity, the higher the BMI value [21]. Increased GH and IGF-1 levels in acromegaly are associated with reduced visceral and subcutaneous fat, but increased intermuscular fat, which could contribute to the development of insulin resistance [37]. Moreover, excess weight is associated with increased systemic inflammation and insulin resistance, which contributes to worsening hypertension and endothelial dysfunction.

### 3.5. Endothelial Dysfunction

Excess GH and IGF-1 in acromegaly cause endothelial dysfunction through various mechanisms, such as increased levels of oxidative stress and reduced anti-oxidant capacity, evidenced by decreased nitric oxide (NO). In addition, it also facilitates vascular wall thickening, reduces the regenerative capacity of the endothelium, and increases the levels of cell adhesion molecules, such as ICAM-1 and VCAM-1 [19,38,39]. These changes contribute to the development of key pathological processes in cardiovascular disease, such as chronic inflammation, atherosclerosis, thrombosis, and vascular remodeling, which significantly increase the risk of serious cardiovascular events, including MI, stroke, and heart failure.

Hyperhomocysteinemia contributes to endothelial dysfunction by promoting oxidative stress, inflammation, altered NO metabolism, a procoagulant state, and direct damage to the endothelium, which accelerates the development of cardiovascular pathologies such as atherosclerosis, thrombosis, and hypertension [20]. Some studies [40], but not all [41], have shown that patients with active acromegaly have higher homocysteine levels compared to those with biochemically controlled acromegaly. Further studies are needed to evaluate the role of elevated homocysteine levels as an independent cardiovascular risk factor for mortality in patients with acromegaly.

### 3.6. Structural and Functional Cardiac Alterations

Chronic GH and IGF-1 exposure in acromegaly affects both myocardial structure and function [42,43,44]. Indeed, acromegaly is often associated with concentric hypertrophy of the left ventricle and, in some cases, of the right ventricle, with an increased thickness of the interventricular septum [43,44]. Specific structural changes in the myocardium have also been reported, including increased myocyte size and interstitial fibrosis in both ventricles [42]. The increased thickening of the intima–media layer of arteries, including coronary arteries, has also been documented in patients with acromegaly compared to healthy controls. This thickening is even more pronounced in patients with active acromegaly than in those with controlled disease [45,46], suggesting an increased risk of atherosclerosis and coronary artery disease in these patients. Functional alterations include left ventricular dysfunction, predominantly diastolic and less frequently systolic, as well as right ventricular systolic dysfunction. In addition, there is an increased incidence of complex ventricular arrhythmias, probably linked to structural changes in the architecture of the left ventricle [9,42,43,44].

### 3.7. Acromegaly Activity and Duration

A prolonged duration and active disease increase the frequency and severity of cardiovascular risks and events [12,47]. Patients with a disease duration of more than 10 years have a three times greater risk of developing cardiac complications compared to those whose disease duration is 5 years or less [47]. Disease duration is the most relevant predictor of left ventricular hypertrophy and systolic dysfunction, whereas patient age is the main predictor of diastolic dysfunction [47]. Disease activity, as estimated by GH and IGF-1 levels, also positively influences the severity of cardiovascular complications [12]. A higher probability of complications such as heart failure and cardiovascular events has been described in men older than 50 years of age [4]. Finally, elderly patients with long-term uncontrolled acromegaly may develop congestive heart failure as the terminal phase of acromegalic cardiomyopathy [48].

## 4. Cardiovascular Diseases

The main cardiovascular diseases associated with acromegaly at diagnosis are coronary artery disease (9.8%), arrhythmias (8.2%), heart failure (7.1%), valvular heart disease (4.9%), stroke (4.3%), and hypertrophic cardiomyopathy (acromegalic cardiomyopathy) (2.3%) [4]. The prevalence of these diseases may vary according to the age at diagnosis, the duration of the disease, the degree of disease activity, and the level of control achieved with medical or surgical treatment. Therefore, the early diagnosis and treatment of acromegaly is essential to reduce the risk of cardiovascular complications. The clinical features and incidence of cardiovascular diseases in acromegaly are summarized in Table 2.

### 4.1. Coronary Heart Disease

Studies on the incidence of coronary heart disease (CHD) in patients with acromegaly are quite limited and have produced conflicting results [12,13,53,54,55,56,57]. As mentioned above, acromegaly is associated with an increased risk of CHD due to the presence of multiple cardiovascular risk factors, such as hypertension, insulin resistance, diabetes, dyslipidemia, endothelial dysfunction, and an increased thickness of the intima–media layer of the coronary arteries [9,11,12]. However, not all studies have shown an increased risk of CHD compared to the general population; some have even pointed out that the risk of coronary events may be low in patients with controlled acromegaly [53,54]. The prevalence of CHD in patients with acromegaly varies widely among studies, from 2.5% in Italy to 12% in France, reflecting considerable heterogeneity [13]. A recent study of 7943 adult patients diagnosed with acromegaly reported a prevalence of CHD and MI at diagnosis of 9.8% and 1.9%, respectively [4]. In comparison, the prevalence of CHD and MI in adults of the general population aged 40 to 79 years is 9.3% and 4.7%, respectively [58]. Considering that acromegaly is usually diagnosed at an average age of 40 years, this suggests that the prevalence of CHD in this condition is relatively low or, at least, does not differ significantly from that observed in the general population.

### 4.2. Cardiac Arrhythmias

Acromegaly is associated with a higher prevalence and incidence of cardiac arrhythmias, especially atrial fibrillation and complex ventricular arrhythmias [4,7,12]. Atrial fibrillation is a common complication in patients with acromegaly, with an estimated prevalence between 4.3% and 7.7% at diagnosis [4,47]. Disease duration, rather than hormone levels, appears to be relevant to these pathological changes [59]. The pathogenesis of arrhythmias in acromegaly appears to be multifactorial, involving the effects of IGF-1 (direct positive inotropic effect on cardiac myocytes by increasing Ca^2^⁺ availability to myofilaments) [60], cardiac structural changes (left ventricular hypertrophy, collagen deposits in cardiac tissues, and fibrosis) [42,43,44], and electrophysiological alterations (increased QT interval variability) [61]. Other arrhythmias such as ectopic beats, paroxysmal supraventricular tachycardia, sick sinus syndrome, ventricular tachycardia, and bundle branch blocks are frequently observed in patients with acromegaly, especially during intense physical exercise [12]. Cardiac arrhythmias are relevant in acromegaly due to their close relationship with increased mortality, mainly through their association with sudden cardiac death.

### 4.3. Heart Failure

Heart failure is a rare but potentially serious complication in patients with acromegaly, frequently associated with acromegalic cardiomyopathy [9,62,63]. In the initial stages, it presents with cardiac hypertrophy and increased contractility and systolic output, features that can be reversed by adequate treatment to normalize GH and IGF-1 levels [64]. However, in advanced stages, the changes in the myocardium become irreversible, evolving toward low cardiac output and systolic and diastolic dysfunction, which severely affects the patient’s life expectancy [62,65,66]. Several factors can contribute to aggravating heart failure in acromegaly, such as hypertension, valvular heart disease (especially mitral and aortic), arrhythmias (such as atrial fibrillation), coronary artery disease, endothelial dysfunction, and diabetes mellitus, which are common in these patients. Early control of acromegaly activity and associated cardiovascular risk factors is essential to prevent the progression of heart failure and improve the prognosis and quality of life of patients.

### 4.4. Valvular Heart Disease

Valvular heart disease is a common and potentially serious complication in acromegaly [67]. It usually depends on the duration of exposure to elevated GH concentrations, with a 19% increase in the odds for each year [68]. It is generally associated with mitral and aortic insufficiency due to valvular degeneration, dilatation of the aortic annulus, and chordae tendineae rupture, with a higher prevalence than in the general population. These valvulopathies generate ventricular overload, hypertrophy, and cardiac dysfunction, exacerbated by the left ventricular hypertrophy typical of acromegaly [69]. The early management of the disease by surgery or pharmacological treatment is essential to prevent the progression of valvular heart disease and improve clinical outcomes, with surgical intervention being necessary in advanced cases [70].

### 4.5. Stroke

According to several studies, the prevalence of stroke in patients with acromegaly does not appear to be significantly higher than in the general population [50,51]. Data from the German Acromegaly Registry, which included 479 patients (56% female, mean age at diagnosis of 46 years, 5549 person-years), indicated that the incidence of stroke was comparable to that in the general population (SIR: 1.17, 95% CI: 0.66–1.93, *p* = 0.61). Furthermore, no association between radiotherapy and stroke was found [50]. In another nationwide, observational, retrospective cohort study conducted in the Korean population (n = 1874 and 9370 age- and sex-matched subjects without acromegaly), the incidence of stroke was similar to that in the general population, with no significant differences observed between the two groups during a mean follow-up period of 7.5 years [51].

### 4.6. Acromegalic Cardiomyopathy

Excess GH and IGF-1 in acromegaly can lead to so-called acromegalic cardiomyopathy, a clinical picture characterized by concentric biventricular hypertrophy and diastolic dysfunction [52,71,72]. Echocardiographic evaluation at the time of diagnosis of acromegaly has indicated a prevalence of left ventricular hypertrophy of 17.8%, with diastolic and systolic dysfunction present in 15.8% and 7.9% of cases, respectively [15]. However, other studies have described a significantly lower prevalence, reaching only 2.3% [4]. Prognosis is conditioned by GH and IGF-1 control. The progression of the disease can lead to heart failure and arrhythmias, associated with increased mortality. Factors such as advancing age, disease duration, increased BMI, and the presence of hypertension or diabetes also aggravate prognosis. However, early treatment, surgical or medical, can improve cardiac function and decrease ventricular hypertrophy [9,12,48,52,71,72].

### 4.7. Cardiovascular Mortality

Cardiovascular mortality is increased in patients with acromegaly [51,73]. A meta-analysis of 16 studies revealed that this condition is associated with a 72% increase in all-cause mortality compared with the general population [74]. Cardiovascular disease is the most frequent comorbidity in acromegaly, affecting approximately 80% of patients and accounting for about 50% of the causes of death in this population [49]. On the other hand, cardiovascular mortality in patients with acromegaly and concomitant diabetes is significantly higher (hazard ratio (HR) of 2.11; 95% CI, 1.09–4.10) compared to patients without diabetes [75]. The main causes of cardiovascular mortality in the population with acromegaly are strongly related to coronary heart disease, stroke, heart failure, and atrial fibrillation, exacerbated by metabolic comorbidities [51].

## 5. Improving Cardiovascular Prognosis in Acromegaly: Therapeutic Strategies and Interventions

Cardiovascular prognosis in acromegaly can be improved by the control of cardiovascular risk factors and comprehensive treatment of cardiovascular comorbidities, as well as the early and appropriate management of acromegaly activity. The main strategies and therapeutic interventions aimed at improving cardiovascular prognosis in acromegaly are summarized in Table 3.

### 5.1. Control of Cardiovascular Risk Factors

#### 5.1.1. Diet and Physical Exercise

As with any patient, it is essential to promote a healthy lifestyle. This includes the promotion of a balanced and adequate diet, and increased regular physical activity adapted to individual conditions, as well as smoking cessation and a reduction in alcohol consumption. It is important to take into account the possible limitations for exercise in patients with acromegaly due to their osteoarticular comorbidity (arthritis and arthropathy, intramuscular fatty infiltration, gait disturbances, and joint pain and dysfunction) [76,77,78], in addition to possible underlying cardiovascular involvement [79,80]. It would be advisable to perform a thorough cardiovascular evaluation before recommending an exercise program and to closely monitor these patients during physical activity. It is essential to adjust exercise programs to minimize the impact on joints, prioritizing low-impact activities such as swimming or cycling [81,82,83,84].

#### 5.1.2. Blood Glucose Control

The pharmacological treatment of diabetes in patients with acromegaly is aimed at counteracting insulin resistance and mitigating the adverse effects of excess GH on glucose metabolism. At present, there are no specific recommendations for the management of diabetes secondary to acromegaly [85]. Recently, however, articles have been published proposing approaches for the optimal treatment of acromegaly-induced diabetes [86,87].

Metformin is a commonly used first-line drug in type 2 diabetes and should also be used in diabetes associated with acromegaly as it improves insulin sensitivity [24,88].

Sodium–glucose cotransporter type 2 (SGLT2) inhibitors have demonstrated efficacy and safety in the treatment of diabetes in patients with acromegaly [89,90]. However, it is advisable to use them in patients who have achieved biochemical control of the disease. This is because active acromegaly may increase the risk of diabetic ketoacidosis (DKA) [91]. In addition, somatostatin receptor ligands, especially pasireotide, can reduce insulin secretion, leading to a state of relative insulin deficiency which, when combined with SGLT2 inhibitors, could increase the risk of DKA in these patients [24,85].

Incretin mimetic drugs such as glucagon-like peptide type 1 (GLP-1) receptor agonists (semaglitude) and dual gastric inhibitory polypeptide (GIP)/GLP-1 receptor co-agonists (tirzepatide) not only improve glycemic control, but also provide additional benefits, such as weight reduction and an improved lipid profile [92], making them promising therapeutic options for patients with acromegaly. However, specific evidence on their efficacy and safety in this population remains limited, highlighting the need for further studies to precisely define their indications and therapeutic guidelines in this patient group.

Given their positive impact on cardiovascular and renal health, in addition to the low risk of hypoglycemia, the use of SGLT2 inhibitors and incretin mimetic drugs should be considered especially in patients with acromegaly at high cardiovascular risk [85].

Insulin may be necessary in patients with poorly controlled diabetes [88]. It can be used in combination with other oral antidiabetics, such as metformin, to optimize glycemic control. However, it is essential to consider the risk of hypoglycemia, especially in patients who have achieved biochemical control of acromegaly. In addition, combination with other treatments, such as somatostatin analogs, requires careful monitoring to maximize efficacy in glycemic control and minimize possible adverse effects [93].

#### 5.1.3. Lipid Profile Management

The specific treatment of dyslipidemia with statins improves the atherogenic lipoprotein profile in acromegaly. In a 3-month, double-blind, placebo-controlled, crossover trial in 11 patients with acromegaly, treatment with atorvastatin 10 mg daily significantly reduced total cholesterol, LDL, VLDL, apolipoprotein B, and the estimated 10-year risk of coronary heart disease, with no significant alterations in HDL or triglycerides [94]. These effects on the lipid profile should be associated with a reduced cardiovascular risk in these patients.

#### 5.1.4. Blood Pressure Control

Antihypertensive treatment in patients with acromegaly is mainly based on the use of angiotensin-converting enzyme inhibitors (ACE inhibitors) and angiotensin II receptor blockers (ARA-II), since they are effective in lowering blood pressure and reducing left ventricular hypertrophy, a frequent complication in this population [95]. As a second-line option, calcium channel blockers and thiazide diuretics can be used as combined or alternative therapy, especially in those patients who do not achieve adequate blood pressure control with ACE inhibitors or ARA-II [34,96].

### 5.2. Control of Acromegaly Activity

Medical treatments used in the management of acromegaly, such as first-generation somatostatin receptor ligands (fg-SRLs) (octreotide and lanreotide), improve disease control but may also cause a slight increase in glycated hemoglobin (HbA1c) and postprandial glucose levels, without significantly affecting fasting glucose. The reduction in insulin and other metabolic parameters suggests that fg-SRLs primarily influence insulin secretion, which may contribute to postprandial hyperglycemia [97]. Overall, it can be concluded that fg-SRLs worsen glucose tolerance and increase HbA1c levels in diabetic patients with acromegaly [97,98,99]. Pasireotide, a second-generation somatostatin receptor ligand (sg-SRL), has been associated with impaired glycemic control due to its increased potency in suppressing endogenous insulin secretion, resulting in increased fasting glucose, glycosylated hemoglobin, and prevalence of type 2 diabetes mellitus [100]. However, pegvisomant exerts a favorable effect on carbohydrate metabolism in patients with acromegaly, including those with diabetes, by decreasing fasting glucose and HbA1c levels, as well as improving insulin sensitivity, which is beneficial for patients both with and without diabetes [101,102,103,104,105]. Finally, dopamine agonists, in particular cabergoline, improve insulin sensitivity and may exert a positive effect on carbohydrate metabolism in patients with acromegaly, including those with diabetes [24]. It has been proposed that the decrease in circulating levels of GH and IGF-I induced by dopaminergic agonists could favor an improvement in glycemic control [106].

Short-term treatment with octreotide long-acting release (LAR) for three months has been associated with a significant decrease in triglyceride levels, the total cholesterol/HDL cholesterol ratio, and Lp(a), while HDL cholesterol (HDL-C) levels increase. These changes were not observed in a slow-release (SR) lanreotide-treated group [107]. Pegvisomant, both as monotherapy and in combination with SSAs, can raise total cholesterol and triglyceride levels, although it reduces Lp(a) levels [108].

The medical treatment of acromegaly using somatostatin analogs, dopaminergic agonists, and GH receptor antagonists is accompanied by an improvement in or the remission of hypertension in some patients [109,110]. The control of GH and IGF-1 levels is associated with significantly lower systolic and diastolic blood pressure, reduced heart rate, and decreased left ventricular mass index. In addition, patients with controlled acromegaly require less antihypertensive medication, indicating that the effective treatment of acromegaly directly correlates with better hypertension control and improved cardiac function [111,112,113,114].

The normalization of GH and IGF-1 levels after transsphenoidal surgery is associated with an improvement in several cardiovascular risk factors, including decreased basal blood glucose, HbA1c, insulin resistance, systolic blood pressure, and triglyceride levels, as well as an increase in HDL cholesterol [115]. It has also been described that 21% of diabetic patients with acromegaly experience the remission of their diabetes after surgery, which is more common in older patients, those who have been cured by surgery, and those with preserved anterior pituitary function [116]. Moreover, an improvement in several parameters of cardiac function has been shown after surgery, most notably a reduction in left ventricular mass and the optimization of diastolic function [117,118,119,120].

Lastly, an unfavorable long-term metabolic profile associated with the use of radiotherapy as adjuvant therapy has been reported in patients who have not achieved adequate control with medical treatment or who have not been cured by transsphenoidal surgery. In this regard, a higher prevalence of obesity, hypertension, and dyslipidemia has been observed compared to patients treated with surgery alone; however, a direct association between radiotherapy and cardiovascular events has yet to be established [121].

### 5.3. Management of Cardiovascular Comorbidities

#### 5.3.1. Arrhythmias

The initial approach to the study and management of arrhythmias in patients with acromegaly includes a diagnostic evaluation with an electrocardiogram and transthoracic echocardiography at the time of diagnosis, with periodic re-evaluation according to the findings and associated cardiovascular comorbidities. Twenty-four-hour Holter monitoring may be useful for the detection of complex ventricular arrhythmias, the prevalence of which is higher in these patients. In addition, it is essential to achieve the normalization of GH and IGF-1 levels by surgery or pharmacological treatment, ensure optimal control of heart failure, and consider the use of devices such as implantable automatic defibrillators or pacemakers, especially in cases of advanced atrioventricular block. It is also important to consider catheter ablation in patients with atrial fibrillation to improve symptoms, quality of life, and ventricular function [9,122,123,124].

#### 5.3.2. Acromegalic Cardiomyopathy: Hypertensive Cardiomyopathy, Dilated Cardiomyopathy, and Heart Failure

The approach to hypertensive heart disease in patients with acromegaly is based on the tight control of hypertension through the use of antihypertensive drugs, reductions in GH and IGF-1 levels through surgery or pharmacological treatment, and the comprehensive management of associated cardiovascular comorbidities, such as diabetes mellitus, dyslipidemia, and sleep apnea [9,13,34,111,120,125]. The management of heart failure in these patients follows standard guidelines for heart failure, including the use of ACE inhibitors, ARA-II, beta-blockers, and mineralocorticoid receptor antagonists [122].

## 6. Conclusions

Acromegaly has a significant impact on the cardiovascular system, which increases the morbidity and mortality prevalent in this population due to complications such as hypertension, arrhythmias, and acromegalic cardiomyopathy. Therefore, the early recognition and management of these complications is essential to improve the prognosis and quality of life of patients. Future research should focus on large-scale, multicenter studies that investigate the long-term effects of different treatment modalities on cardiovascular health in patients with acromegaly. Additionally, specific studies exploring the genetic and biomarker profiles that predict cardiovascular complications could lead to personalized approaches in management. The normalization of GH and IGF-1 levels by surgical and/or medical treatment can partially reverse cardiovascular alterations, although some complications may persist in advanced cases. Clinicians should prioritize the routine monitoring of GH and IGF-1 levels, alongside regular cardiovascular assessments, to detect complications early. Implementing standardized protocols for cardiovascular evaluation in patients with acromegaly can help in stratifying risk and tailoring treatment plans effectively. Furthermore, ongoing education for healthcare professionals regarding the cardiovascular risks associated with acromegaly is critical in enhancing patient outcomes. Likewise, it is essential to control traditional cardiovascular risk factors and perform a comprehensive cardiovascular evaluation to prevent adverse events.

## Figures and Tables

**Table 1:** Effects of acromegaly on cardiovascular risk, pathogenic mechanisms, and associated clinical consequences.

Effects on Cardiovascular Risk	Pathogenic Mechanism in Acromegaly	Clinical Consequences
Effects on carbohydrate metabolism	Stimulation of hepatic gluconeogenesis and reduction in insulin sensitivity by GH. May also affect insulin secretion by beta-cell dysfunction	Hyperglycemia Hyperinsulinism Increased risk of prediabetes (26–41%), and type 2 diabetes (22.3–76.8%) [4,6,10,14]
Effects on lipid metabolism	Elevated total cholesterol, LDL cholesterol, VLDL cholesterol, triglycerides, and lipoprotein (a) with decreased HDL cholesterol. Increased small and dense LDL particles	Dyslipidemia (up to 61%) Accelerated atherosclerosis [10,12,14,16,17]
Effects on blood pressure	Expansion of plasma volume, promotion of sodium and water retention, and endothelial dysfunction	Hypertension (18–77%) Increased risk of cardiovascular disease [4,10,13,14,18]
Effects on endothelial function	Increased oxidative stress and decreased anti-oxidant capacity with reduced nitric oxide. Promotes vascular thickening, decreases endothelial regeneration, and increases cell adhesion molecules (ICAM-1, VCAM-1)	Endothelial dysfunction Increased risk of cardiovascular events [4,10,12,19,20]
Effects on body weight	Increased intermuscular fat Systemic inflammation Insulin resistance	Obesity (more than 40%) Worsening hypertension and endothelial dysfunction Deterioration of endothelial dysfunction [10,12,14,21]
Effects on cardiac structure and function	Concentric hypertrophy of left ventricle Increased myocyte size and interstitial fibrosis in both ventricles Thickening of intima–media layer of arteries, including coronary arteries	Increased risk of atherosclerosis and coronary artery disease Left ventricular dysfunction, predominantly diastolic Increased incidence of complex ventricular arrhythmias [4,10,12,14]
Effect of activity and duration of acromegaly	Increase in frequency and severity of cardiovascular risks and events owing to prolonged duration and active disease	Increased frequency of acromegalic cardiomyopathy [4,10,12,14]

**Table 2:** Clinical features and incidence of cardiovascular diseases in acromegaly.

Cardiovascular Diseases	Clinical Features	Prevalence
Coronary artery disease (CHD)	Similar prevalence to that in the general population	CHD (9.8%) and myocardial infarction (1.9%) at diagnosis [4]
Arrhythmia	Especially atrial fibrillation and complex ventricular arrhythmias Close relationship with increased mortality, mainly through its association with sudden cardiac death	Atrial fibrillation (4.3–7.7%) at diagnosis [4,12]
Heart failure	Frequently associated with acromegalic cardiomyopathy	7.1% [4]
Valvular heart disease	Mitral and aortic insufficiency due to valvular degeneration, dilatation of the aortic annulus, and chordae tendineae rupture	4.9% [4,49]
Stroke	Similar prevalence to that in the general population	4.3% [50,51]
Acromegalic cardiomyopathy	Concentric biventricular hypertrophy and diastolic dysfunction	2.3% [4,52]
Cardiovascular mortality	Cardiovascular mortality increased compared with the general population Strongly related to coronary heart disease, stroke, heart failure, and atrial fibrillation	Standardized mortality ratio (SMR) of 2.95 (95% CI: 2.35–3.55) [49,51]

**Table 3:** Main strategies and therapeutic interventions aimed at improving cardiovascular prognosis in acromegaly.

Strategy/Intervention	Description	Objective
Control of cardiovascular risk factors		
Diet and physical exercise	A healthy lifestyle can be promoted by a balanced and adequate diet and increased regular physical activity according to tolerance	Improve weight control and cardiovascular health
Blood glucose control	Metformin is the first-line drug SGLT2 inhibitors are effective and safe in controlled acromegaly GLP-1 receptor agonists are promising therapeutic options for patients with acromegaly Insulin may be necessary in patients with poorly controlled diabetes	Improve insulin sensitivity and glycemic control
Lipid profile management	Statins are used	Improve atherogenic lipoprotein profile in acromegaly
Blood pressure control	First-line drugs: angiotensin-converting enzyme inhibitors and angiotensin II receptor blockers Second-line drugs: calcium channel blockers and thiazide diuretics	Lower blood pressure and reduce left ventricular hypertrophy
Control of acromegaly activity		
Blood glucose control	Pasireotide may worsen glycemic control Pegvisomant improves insulin sensitivity Cabergoline agonists may improve carbohydrate metabolism Transsphenoidal surgery is associated with a decrease in basal blood glucose, HbA1c, and insulin resistance, with a remission of diabetes in 21% of patients	Improve cardiovascular risk profile
Lipid profile management	Octreotide LAR decreases triglyceride levels, the total cholesterol/HDL cholesterol ratio, and Lp(a), and increases HDL cholesterol levels Pegvisomant can raise total cholesterol and triglyceride levels, although it reduces Lp(a) levels Transsphenoidal surgery is associated with a decrease in triglyceride levels and an increase in HDL cholesterol	
Hypertension	The medical treatment of acromegaly using somatostatin analogs, dopaminergic agonists, and GH receptor antagonists is accompanied by improvement in or the remission of hypertension in some patients Transsphenoidal surgery is associated with a decrease in blood pressure	
Management of cardiovascular comorbidities		
Arrythmias	Electrocardiogram and transthoracic echocardiography are used at diagnosis Twenty-four-hour Holter monitoring is used for the detection of complex ventricular arrhythmias Implantable automatic defibrillators or pacemakers are used, especially in cases of advanced atrioventricular block Catheter ablation is considered in patients with atrial fibrillation	Early detection and adequate management of cardiovascular complications Improve symptoms and increase quality of life and survival
Acromegalic cardiomyopathy	The tight control of hypertension, diabetes mellitus, dyslipidemia and sleep apnea can lead to improvement The adequate management of heart failure following updated standard guidelines is necessary	

## Data Availability

No original data are associated with this manuscript.
